# Uncovering environmental pollutants affecting Atlantic cod growth with machine learning

**DOI:** 10.1093/bib/bbaf631.047

**Published:** 2025-12-12

**Authors:** Ayumu Muta, Kazuhiro Takemoto, Midori Iida

**Affiliations:** Department of Physics and Information Technology, Kyushu Institute of Technology, Iizuka, Fukuoka, Japan; Department of Bioscience and Bioinformatics, Kyushu Institute of Technology, Iizuka, Fukuoka, Japan; Data Science and AI Research Center, Kyushu Institute of Technology, Iizuka, Fukuoka, Japan; Department of Physics and Information Technology, Kyushu Institute of Technology, Iizuka, Fukuoka, Japan

## Abstract

**Aim:**

Water and fishery resources are essential for human life and regional economies. However, marine ecosystems face increasing pressures from human activities such as urbanization, agriculture, and climate change. Although the effects of rising sea temperatures on fish growth have been well documented [1], the impacts of chemical pollutants, including pesticides and heavy metals, remain less understood. This study aimed to identify environmental pollutants and oceanographic factors influencing Atlantic cod (*Gadus morhua*) growth using interpretable machine-learning methods.

**Methods:**

Comprehensive datasets of environmental pollutants and oceanographic parameters from the North Atlantic were compiled. Random forest regression combined with SHAP (SHapley Additive exPlanations) was used to quantify variable importance.

**Results:**

Zinc (Zn), lead (Pb), and chromium (Cr) exhibited significant negative contributions to predicted body length, suggesting potential growth inhibition associated with these metals. In contrast, mercury (Hg) showed a positive association with body size, suggesting bioaccumulation consistent with previous findings. Furthermore, dissolved oxygen, salinity, and temperature were also identified as important predictors.

**Conclusion:**

These findings indicate that both chemical pollutants and oceanographic parameters influence cod growth. Recognizing the importance of these two qualitatively different types of environmental factors can contribute to more comprehensive approaches in ecosystem-based fisheries management and in the environmental risk assessment and management of marine pollutants.

**References:**

[1] Pörtner H.O., Knust R. ‘Oxygen- and capacity-limited thermal tolerance: Linking ecology and physiology.’ Annual Review of Physiology 2010; 72:61–90.

